# Novel evidence that the ABO blood group shapes erythropoiesis and results in higher hematocrit for blood group B carriers

**DOI:** 10.1038/s41375-023-01858-4

**Published:** 2023-02-28

**Authors:** Romy Kronstein-Wiedemann, Sarah Blecher, Madeleine Teichert, Laura Schmidt, Jessica Thiel, Markus M. Müller, Jörn Lausen, Richard Schäfer, Torsten Tonn

**Affiliations:** 1 Laboratory for Experimental Transfusion Medicine, Transfusion Medicine, Med. Faculty Carl Gustav Carus, Technische Universität Dresden, Dresden, Germany; 2 German Red Cross Blood Donation Service North-East, Institute for Transfusion Medicine, Dresden, Germany; 3 German Red Cross Blood Donation Service Baden-Württemberg/Hessen, Institute for Transfusion Medicine and Immunohematology, Kassel, Germany; 4 Department of Genetics of Eukaryotes, Institute of Biomedical Genetics, University of Stuttgart, Stuttgart, Germany; 5 German Red Cross Blood Donation Service Baden-Württemberg/Hessen, Institute for Transfusion Medicine and Immunohematology, Goethe University Hospital Frankfurt/M, Frankfurt/M, Germany; 6 Institute for Transfusion Medicine and Gene Therapy Medical Center - University of Freiburg, Freiburg, Germany

**Keywords:** Haematopoietic stem cells, Stem-cell research

## Abstract

The ABO blood group (BG) system is of great importance for blood transfusion and organ transplantation. Since the same transcription factors (TFs) and microRNAs (miRNAs) govern the expression of ABO BG antigens and regulate erythropoiesis, we hypothesized functional connections between both processes. We found significantly higher hemoglobin and hematocrit values in BG B blood donors compared to BG A. Furthermore, we observed that erythropoiesis in BG B hematopoietic stem/progenitor cells (HSPCs) was accelerated compared to BG A HSPCs. Specifically, BG B HSPCs yielded more lineage-specific progenitors in a shorter time (B: 31.3 ± 2.2% vs. A: 22.5 ± 3.0%). Moreover, non-BG A individuals exhibited more terminally differentiated RBCs with higher enucleation rates containing more hemoglobin compared to BG A. Additionally, we detected increased levels of miRNA-215-5p and -182-5p and decreased expression of their target TFs RUNX1 and HES-1 mRNAs in erythroid BG B precursor cells compared to BG A. This highlights the important roles of these factors for the disappearance of differentiation-specific glycan antigens and the appearance of cancer-specific glycan antigens. Our work contributes to a deeper understanding of erythropoiesis gene regulatory networks and identifies its interference with BG-specific gene expression regulations particularly in diseases, where ABO BGs determine treatment susceptibility and disease progression.

## Introduction

The steady-state supply of mature red blood cells (RBCs) requires the release of more than 2 million reticulocytes every second from the bone marrow (BM) into the blood stream. This is achieved by the fine-tuned process of erythropoiesis [1] being mainly located in the erythroblastic islands of the BM. Erythropoiesis involves interconnected steps from the attachment of erythroblasts to macrophages, over nuclear reorganization, the programmed destruction of organelles and surface membrane protein sorting [2]. The resulting reticulocytes complete their maturation within 1–2 days in the blood stream [3]. Throughout erythroid maturation, surface markers as well as the cytoskeleton structure continuously evolve [4] due to rearrangements of the cell membrane and active exocytosis [5].

Complex gene regulatory networks that rely on transcriptional and post-transcriptional circuits orchestrate erythropoiesis. Transcription factors (TFs) and microRNAs (miRNAs) are involved in determining the correct temporal activation, or repression, of specific hematopoietic transcriptional programs during both hematopoietic stem and progenitor cells (HSPCs) maintenance and their lineage-specific differentiation and function [6]. GATA-binding factor (GATA)-1, GATA-2 and zinc finger TFs play a central role in erythroid differentiation by binding to functionally important DNA regulation sequences in erythroid and megakaryocytic-specific genes [7]. Specifically, GATA-1 is necessary for the survival and terminal differentiation of erythroid progenitors (ERPs), whereas GATA-2 regulates the maintenance and proliferation of HSPCs [8].

In addition to binding to DNA, specific proteins interact physically with GATA-1 including T-cell acute lymphocytic leukemia protein 1 (TAL-1), erythroid Kruppel-like factor (EKLF)/ specificity protein-1 (SP1) [9], E1A-associated protein/ CREB-binding protein (p300/CBP) [10], and friends-of-GATA-1 (FOG-1) [11]. These are crucial factors for HSPCs development in the embryo and for gene regulation during their erythroid/megakaryocytic differentiation [7, 12, 13]. In contrast, other TFs such as Runt-related TF 1 (RUNX-1) [14] and hairy and Enhancer of Split-1 (HES-1) [15] are important for the repression of erythroid genes and the establishment of the megakaryocytic gene expression program.

Additional important regulators of erythropoiesis are miRNAs that govern posttranscriptional gene expression by translational repression or by destabilizing target transcripts [16]. Besides chromatin modifications including histone acetylation and DNA methylation [17], miRNAs modulate hematopoietic differentiation, proliferation, as well as self-renewal and lineage-specific potential of hematopoietic cells by targeting the expression of TFs and genes involved in the regulation of cell cycling and proliferation [18].

The ABO system is the clinically most relevant BG system, and is defined by the ABO BG antigens that can be detected already during early erythropoiesis [19, 20]. The antigens in the ABO system are oligosaccharides, and their synthesis depends on the biochemical reactions catalyzed by two glycosyltransferases (A- and B-transferases) coded by the functional A and B alleles at the ABO genetic locus [21, 22]. Since ABO BG antigens expression is associated with the terminal differentiation of ERPs [21], it can be hypothesized that the switch-on mechanisms for both ABO genes expression and erythropoiesis are governed by similar regulatory pathways. Specifically, the binding of SP1 or SP1-like protein(s) to the ABO promoter may play an important role for its functionality in both erythroid and epithelial cell lineages [23].

Furthermore, binding sites for both GATA-1 and SP1 are located at the promoter- and enhancer-regions of genes expressed in erythroid cells where they appear to interact synergistically [9]. Moreover, an erythroid cell-specific regulatory element, referred to as the +5·8‐kb site, was identified in the first intron of the ABO gene, and its regulatory activity depends upon the binding of GATA-1 or -2, and RUNX-1 [24, 25].

In addition to these physiological processes, profound changes in BG antigens expression are also observed in pathological conditions such as tumorigenesis [26–28]. Reduced expression, or complete deletion of A/B antigens was reported in primary lung, bladder, and colorectal tissue carcinomas [29–31]. Furthermore, the loss of A, B or H antigens from the surface of RBCs can be seen in hematological malignancies. Thus, the loss of ABO BG antigens expression in an RBC population derived from a malignant HSC clone could be an indicator of genetic changes during oncogenesis on the stem/progenitor cell level [32].

Since the expression of ABO BG antigens and erythropoiesis are governed by identical TFs, we hypothesized the existence of a functional connection between both processes. Additionally, we previously identified miRNAs that play a critical role in the regulation of the ABO BG system [22]. Interestingly, various miRNAs differ in their expression patterns depending on the BG genotype. Some of these miRNAs, such as miR-331-3p, have potential binding sites within both the 3′UTR of the ABO glycosyltransferases genes and the 3′UTR of several TF genes, like SP1 and RUNX-1, which regulate both erythropoiesis and ABO BG antigens expression. We found that overexpression of miR-331-3p leads to downregulation of BG A antigen expression by the simultaneous targeting of TF SP1, thus rendering SP1 to be incapable of binding to the promoter of the ABO gene [22].

Based on these observations, we aimed in this study to investigate, whether erythropoiesis is (co-) regulated by ABH BG antigen expression.

## Methods

### Determination of hemoglobin content and hematocrit value

Three milliliters whole blood from healthy donors were collected in S-Monovettes EDTA K3 (Sarstedt, Nürnbrecht, Germany) by venipuncture using sterile disposable Safety-Multifly®- Needle 21G (Sarstedt). Determination of hemoglobin contents and hematocrit values were performed with Sysmex XN 1000 (Sysmex Deutschland GmbH, Norderstedt, Germany).

### Statistical analyses

Data were analyzed with GraphPad Prism 5 (San Diego, USA) using student *t* test (*n* = 3) or Mann–Whitney *U* test (*n* ≥ 4). *P* values of less than 0.05 were considered significant. Tests were performed two-sided. Data presented as mean and error bars depict the standard error of the mean (s.e.m.). Independent experimental repeats from at least 13 donors (except BG ABO*A1.01B1.01 with only five donors) were performed. No data were excluded from analysis. No randomization or blinding was used in any experiments. qPCR analysis could not be performed for all samples on day 5, due to there were not always enough samples available on this day.

### Study approval and Ethics

G-CSF-mobilized peripheral blood samples were provided by the German Red Cross Donation Service Baden-Württemberg/Hessen, Institute for Transfusion Medicine and Immunohematology, Frankfurt and were used in accordance with the guidelines approved by the Ethics Committee of the Technical University of Dresden. Informed consent was obtained from all donors.

Other experimental procedures are available in supporting information (SI) Methods.

## Results

### RBCs from individuals with BG B carry more hemoglobin than RBCs of non-B BGs

**Fig. 1:**
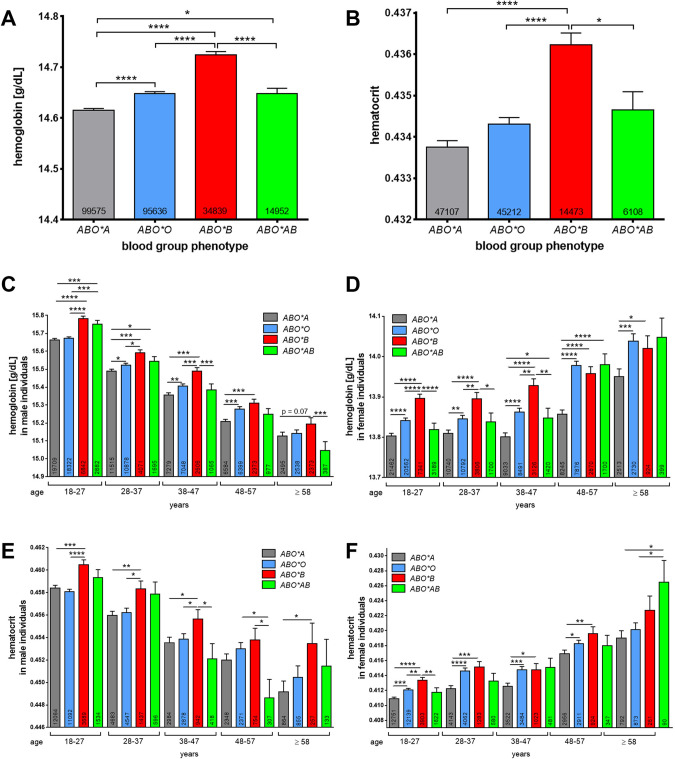
Large-scale analysis of hemoglobin content and hematocrit in RBCs from blood donors focusing on BG distributions. Analysis of the Hb content (**A**) and hematocrit (**B**) from 245.002 first time blood donors carrying different ABO BG antigens. Analysis of the Hb content from first time male (**C**) and female (**D**) blood donors carrying different ABO BG antigens depending from the age of the donors. Analysis of hematocrit values from first time male (**E**) and female (**F**) blood donors carrying different ABO BG antigens depending from the age of the donors. The number of analyzed donors is indicated in the corresponding bars. Data are presented as mean ± SEM (standard deviation error). Tests were performed two-sided. *P < 0.05; ***P < 0.001 (Mann–Whitney *U* test).

To assess the clinical relevance of a possible link of BG expression to erythropoiesis, we conducted a large-scale (*N* = 245,002) analysis from first time blood donors carrying different ABO BGs. In this cohort we analyzed the in vivo hemoglobin (Hb) content and hematocrit in RBCs. Donors of BG ABO*B showed significantly higher Hb contents and hematocrit values than individuals of non-B BGs with the lowest values in individuals of BG ABO*A (Fig. 1A, B). These differences were independently of age and sex of the donors, although both Hb contents and hematocrit values decreased continuously with age in men in contrast to women (Fig. 1C–F; Supplemental Tables S1–S4).

### The differentiation and proliferation potential is reduced in HSPCs from BG A individuals compared to HSPCs from donors with other BGs

**Fig. 2:**
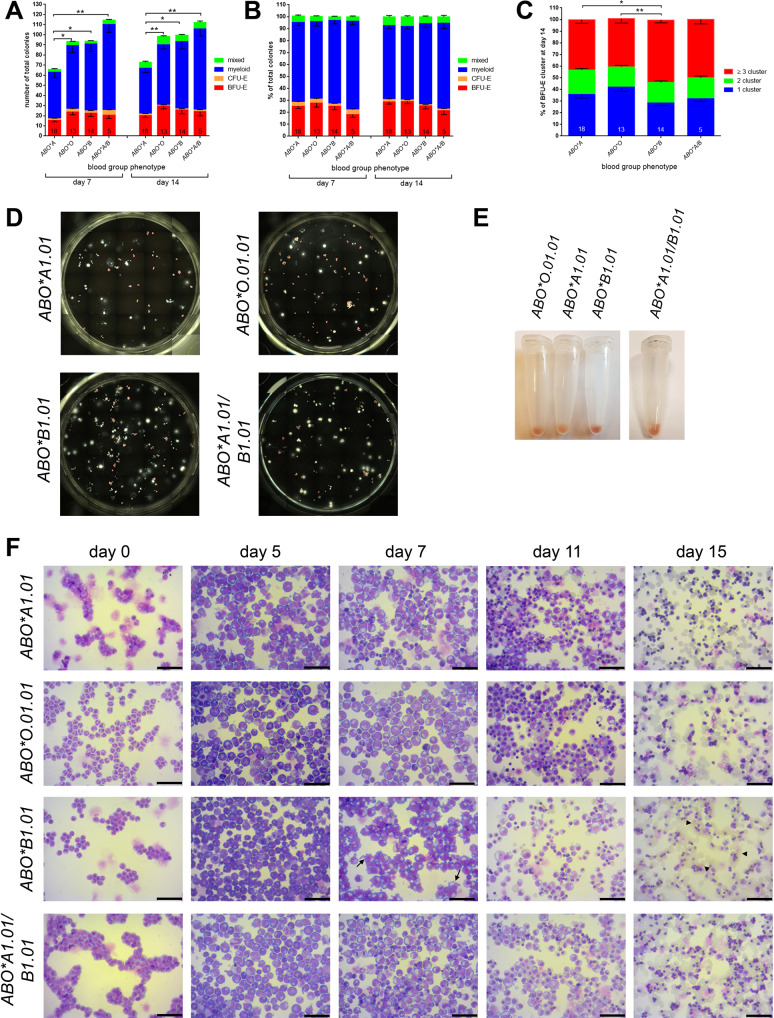
Impact of the ABO BG on the in vitro differentiation and proliferation potential of HSPCs. **A** Depicted are the numbers of total colonies in dependence of the ABO BG phenotype derived from 300 HSPCs per well. Colony formation was analyzed after 7 and 14 days. **B** Percentage of the type of colonies within the total number of colonies. **C** Percentage of the number of clusters (1, 2 or ≥ 3) within the BFU-E colonies at day 14. **A**–**C** The number of analyzed donors is indicated in the corresponding bars. Data are presented as mean ± SEM. Tests were performed two-sided. *P < 0.05; **P < 0.01 (Mann–Whitney *U* test). **D** One representive well per BG phenotype of an in vitro colony forming unit assay at day 14 is shown. **E** Cell pellets of HSCs carrying differents ABO BG antigens in vitro differentiated to erythroid cells for 7 days. **F** Morphological analysis of HSCs carrying differents ABO BG antigens during in vitro erythropoiesis. Cells were stained with Giemsa solution (bar = 50 µm). Arrows indicate “membrane blebbing” and arrow heads indicate enucleated mature RBCs. **E**, **F** One representative sample per BG phenotype is shown.

We next examined if the ABO BG would affect the in vitro differentiation and proliferation potential of HSPCs. Specifically, we tested the colony formation potential of CD34 + HSPCs from donors with different ABO BGs. In order to exclude variations in typical ABO polymorphisms [33] we performed PCR analysis of the 43 bp enhancer tandem elements in the 5′ area of the gene and the A1-specific 36 bp deletion from all samples (supplemental Table S5). Numbers of lineage-specific progenitors derived from HSPCs of BG ABO*O.01.01, ABO*B1.01 and ABO*A1.01/B1.01 were increased compared to HSPCs from BG ABO*A1.01 (Fig. 2A). However, the percentage of lineage-specific progenitor subtypes within the total number of colonies remained unaffected by the BG phenotype (Fig. 2B, supplemental Table S6). Further, larger burst forming unit-erythroids (BFU-E) could be generated from HSPCs of BG ABO*B1.01 compared to HSPCs from the other BGs (Fig. 2C, D).

### BG B hematopoietic cells display accelerated and more efficient erythropoiesis

During erythropoiesis, HSPCs undergo a series of morphological changes including a decrease in cell size, the onset of Hb formation, a gradual reduction of cell organelles, and nuclear reorganization. We quantified Hb formation at day seven of in vitro differentiation of HSPCs by monitoring the intensity of the red coloration of the cell pellets (Fig. 2E). While the pellets of the cells from donors of BG ABO*A1.01 showed only a slight reddish color, we noted a marked increase in Hb formation in hematopoietic cells from donors of the other BG phenotypes with the strongest Hb production in cells of BG ABO*B1.01.

The morphology changes confirmed the accelerated erythropoiesis of HSPCs from BG ABO*B1.01 carrying donors compared to HSPCs from donors of the other BGs (Fig. 2F). At day 5 in this group, we detected smaller sized cells, indicating a higher number of basophilic erythroblasts. At day 7 of erythropoiesis, we observed the typical “membrane blebbing” only in samples from donors of BG ABO*B1.01 (Fig. 2F, arrows). Furthermore, the terminal differentiation into mature RBCs was enhanced in samples from donors of BG ABO*B1.01 compared to samples from donors of the other BG phenotypes (Fig. 2F, arrowheads).

### BG ABO*B1.01 HSPCs produce more erythroid precursor subsets and enucleated RBCs

**Fig. 3:**
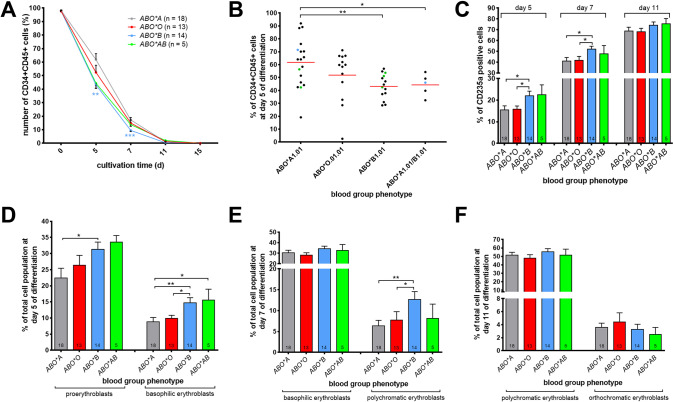
Impact of ABO BG on expression of erythroid specific surface markers during erythroid differentiation. Cells were cultivated for 15 days in erythroid differentiation medium and stained at indicated time points with fluorochrom labeled antibodies against CD34, CD45, CD235a, CD71 and CD36. Analysis was performed by flow cytometry. **A** Percentage of expression of the HSC markers CD34 and CD45 during erythropoiesis in dependence of the ABO BG phenotype. **B** Percentage of the CD34 and CD45 expression on day 5. Each dot represents one donor. Green dots indicate homozygous donors and the blue dots indicate donors with BG ABO*A2.01 and *A2.01/B1.01*, respectively. **C** Percentage of CD235a positive cells within the total cell number at indicated time points of differentiation. (D-F) Percentage of different cell subtypes at day 5 (**D**), day 7 (**E**), and day 11 (**F**) of erythroid differentiation. Proerythroblasts were defined as CD34^neg^/CD45^pos^/CD71^high^/CD36^high^/CD235a^neg^. Basophilic erythroblasts are CD34^neg^/ CD45^low^/CD71^high^/CD36^high^/CD235a^pos^. Polychromatic erythroblasts were assigned to CD34^neg^/ CD45^neg^/CD71^pos^/CD36^pos^/CD235a^high^ and orthochromatic erythroblasts to CD34^neg^/CD45^neg^/ CD71^pos^/CD36^neg^/CD235a^high^. **C-F** The number of analyzed donors is indicated in the corresponding bars. Data are presented as mean ± SEM. Tests were performed two-sided. *P < 0.05; **P < 0.01 (Mann–Whitney *U* test).

Erythroid differentiation is associated with substantial changes in surface marker expression. Thus, we examined the hematopoietic and erythroid markers CD34, CD45, CD71, CD36, and CD235a by flow cytometric analyses (Fig. 3). The expression of the HSPC marker CD34 and the non-erythroid lineage marker CD45 were down-regulated during erythropoiesis, however significantly faster in cells from donors of BG ABO*B1.01 as compared to cells from donors of BG ABO*A1.01 (Fig. 3A, B). Moreover, the specific erythroid marker CD235a (glycophorin A) was upregulated, with the number of CD235a-positive cells in donors of BG ABO*B1.01 on days 5 and 7 being significantly higher compared to donors of BG ABO*A1.01 (Fig. 3C). We then assessed the erythropoiesis of different hematopoietic progenitor subpopulations according to the expression patterns of their surface marker profiles. Proerythroblasts were defined as CD34neg/CD45pos/CD71high/CD36high/CD235aneg, basophilic erythroblasts as CD34neg/ CD45low/CD71high/CD36high/CD235apos, polychromatic erythroblasts as CD34neg/CD45neg/ CD71pos/CD36pos/CD235ahigh, and orthochromatic erythroblasts as CD34neg/CD45neg/ CD71pos/CD36neg/CD235ahigh. At day 5 of erythroid differentiation, we found a significantly higher number of proerythroblasts and basophilic erythroblasts in the cell culture of HSPCs of BG ABO*B1.01 (mean values ~31.8% and 13.8%) compared to BG ABO*A1.01 (mean values ~20.1% and 7.0%) (Fig. 3D), which was almost equalized until day 7 (Fig. 3E). In contrast, at day 7 the number of polychromatic erythroblasts in the cell culture of HSPCs from donors of BG ABO*B1.01 (mean value ~9.8%) were almost twice the HSPCs from donors of BG ABO*A1.01 (mean value ~4.5%) (Fig. 3E). From day 11, no differences in surface marker expression and thus, the hematopoietic subpopulations, were seen between the BGs (Fig. 3F). However, at day 15 we harvested 30–50% enucleated cells derived from HSPCs of BG ABO*B1.01, whereas the number of enucleated cells from HSPCs of BG ABO*A1.01 was only 10–30%.

### The transcriptional regulation of erythropoiesis is determined by the ABO BG

**Fig. 4:**
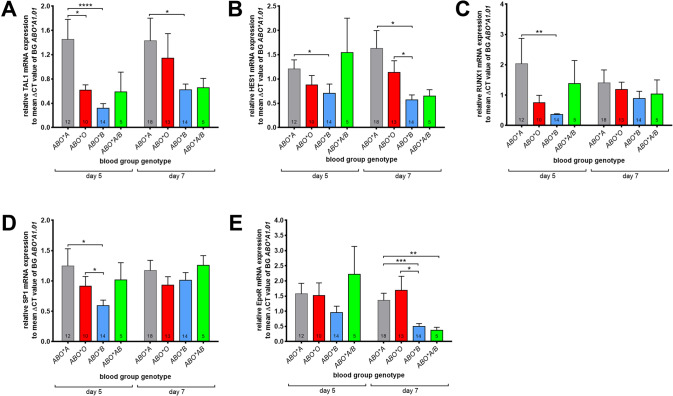
The transcriptional regulation of erythropoiesis depends on the BG phenotype. Relative expression of *TAL1* (**A**), *HES1* (**B**), *RUNX1* (**C**), *SP1* (**D**), and *EpoR* mRNA (**E**) to the mean value of ΔCT from cells carrying BG ABO*A1.01. Quantitative PCR was performed at day 5 and day 7 of erythroid differentiation. Relative fold changes in expression (normalized to *GAPDH*) were calculated by the ΔΔCT method and values are expressed as 2-ΔΔCT. The number of analyzed donors is indicated in the corresponding bars. Data are presented as mean ± SEM. Tests were performed two-sided. *P < 0.05; **P < 0.01; ***P < 0.001 (Mann–Whitney *U* test).

To investigate how the molecular regulation of the erythropoiesis is determined by the ABO BGs we next analyzed the mRNA and protein expression levels of key erythropoiesis TFs. At day 5 of erythroid differentiation the mRNA expression levels of *TAL-1*, *HES-1* (both by about 60–70%), *RUNX1*, and *SP1* (both by about 40–50%) were strongly decreased in ERPs from donors of BG ABO*B1.01 compared to BG ABO*A1.01 (Fig.4A–D). Furthermore, the expression of the *EPOR* mRNA was decreased in ERPs from donors of BG ABO*B1.01 and ABO*A1.01/B1.01 (by about 60–70%) compared to the non-B BGs (Fig. 4E) at day 7 of HSPC differentiation. In contrast, the mRNA expression levels of *GATA-1*, its binding partners *p300* and *glucocorticoid receptor* (GR) was unaffected by the BG types (Supplemental Fig. S1).

**Fig. 5:**
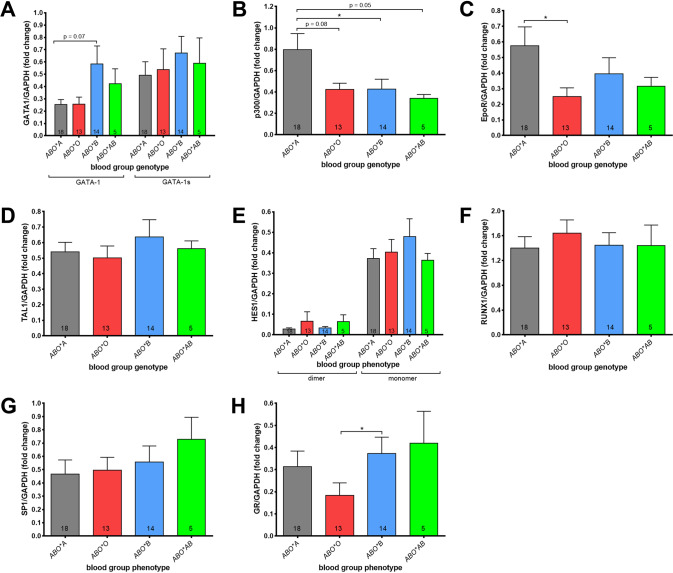
GATA-1 showed an accelerated upregulation during erythropoiesis of HSCs carrying BG B. Quantification of GATA-1 (**A**), p300 (**B**), EpoR (**C**), TAL1 (**D**), HES1 (**E**), RUNX1 (**F**), SP1 (**G**), and glucocorticoid receptor (**H**) protein expression by western blot analysis at day 7 of in vitro erythropoiesis. Expression was normalized to GAPDH protein expression as endogeneous loading control (fold change). The number of analyzed donors is indicated in the corresponding bars. Data are presented as mean ± SEM. Tests were performed two-sided. *P < 0.05 (Mann–Whitney *U* test).

Analyses of protein expression (Fig. 5, Supplemental Fig. S2) showed a tendency towards higher expression of the central TF GATA-1 in ERPs from donors carrying BG ABO*B1.01 compared to ERPs from the other BGs at day 7 of differentiation (Fig. 5A). In contrast, at this time point, the p300 protein was markedly increased in ERPs from donors of BG ABO*A1.01 compared to the other BGs with the greatest difference to cells from BG ABO*B1.01 (Fig. 5B). Furthermore, the expression of the EPOR, which is crucial for the proliferation and survival of progenitors, but not for commitment or differentiation [34], was enhanced in cells from donors of BG ABO*A1.01 as compared to the other BG types (Fig. 5C). Expression of GR protein was reduced in ERPs from donors of BG ABO*O1.01, too (Fig. 5D). There was also a substantial heterogeneity in the differentiation state at day 7, as we measured no differences in the expression of TAL1, HES1, RUNX1, and SP1 (Fig. 5E–H). Therefore, we further evaluated correlations of the TF expressions with the number of CD34+ positive cells and proerythroblasts, except for BG ABO*A1.01/B1.01 due to the low sample numbers. The decrease of CD34+ cells and the increase of proerythroblasts at day 5 of differentiation was linear in all BG samples with a measure of determination coefficient of R^2^ = 0.61 (ABO*B1.01) to 0.87 (ABO*A1.01) (Supplemental Fig. S3A). Although we found a higher expression of GATA-1 in cells carrying BG ABO*B1.01 at day 7 of differentiation (Fig. 5A), there was a strong negative correlation between the number of proerythroblasts at day 5 and the expression of GATA-1 (Supplemental Fig. S3B, Supplemental Table S7). In contrast, we noted a positive correlation in cells carrying BG ABO*A1.01. We also found strong negative correlations between the expression of TAL-1, HES-1 monomer, RUNX-1, and SP1 at day 7 of differentiation and the number of proerythroblasts at day 5, respectively, notably only during erythropoiesis of cells from BG ABO*B1.01 (Supplemental Fig. S3B, Supplemental Table S7). Moreover, we observed a moderate correlation between the expression of EPOR (*R*^2^ = 0.32) and the number of proerythroblasts in the ERP cultures from donors of BG ABO*B1.01, but we could not detect a correlation to the p300 and GR protein expression (Supplemental Table S7). This is in stark contrast to BG ABO*A1.01 and ABO*O1.01, where we found a clear positive correlation of GR protein expression and the number of proerythroblasts (Supplemental Table S7). Furthermore, only in cells of BG ABO*A1.01 the expression of GATA-1 protein positively correlated with the expression of GR protein at day 7 of erythroid differentiation.

### Differential miRNA expression patterns during erythropoiesis from cells of different ABO BG types

**Fig. 6:**
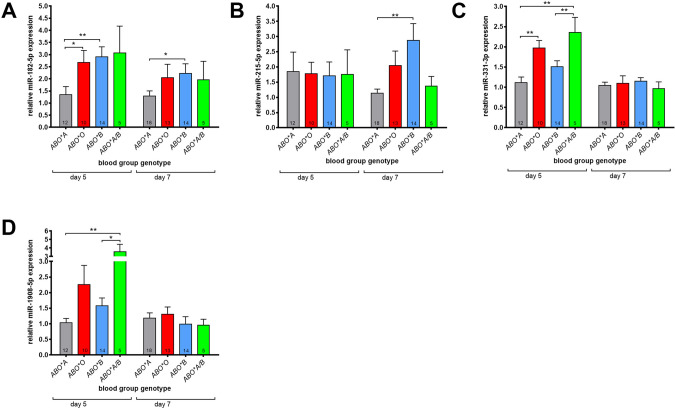
Differential miRNA expression pattern during erythropoiesis from cells of different ABO BG genotypes. Relative expression of miR-182-5p (**A**), miR-215-5p (**B**), miR-331-3p (**C**), and miR-1908-5p (**D**) to the mean value of ΔCT from cells carrying BG ABO*A1.01. Quantitative PCR was performed at day 5 and day 7 of erythroid differentiation. Relative fold changes in expression (normalized to U6) were calculated by the ΔΔCT method and values are expressed as 2-ΔΔCT. The number of analyzed donors is indicated in the corresponding bars. Data are presented as mean ± SEM. Tests were performed two-sided. *P < 0.05; **P < 0.01 (Mann–Whitney *U* test).

Since it was reported that *HES-1* mRNA is a target of miR-182-5p [15] and miR-215-5p targets *RUNX-1* mRNA [35], we evaluated the expression levels of these miRNAs. We found twofold higher expression levels of miR-182-5p on both day 5 and 7 of erythroid differentiation in ERPs of BG ABO*B1.01 as compared to BG ABO*A1.01 (Fig. 6A). The expression of miR-215-5p (Fig. 6B) only increased at day 7 in cells from ABO*B1.01 (by about 2.3-fold), and the expressions of miR-331-3p and miR-1908-5p were transiently higher at day 5 in BG ABO*0.01.01 and BG ABO*A1.01./B1.01 individuals and equalized on day 7 (Fig. 6C, D).

### Inhibition of RUNX1 downregulates BG A antigen expression and accelerates erythropoiesis

**Fig. 7:**
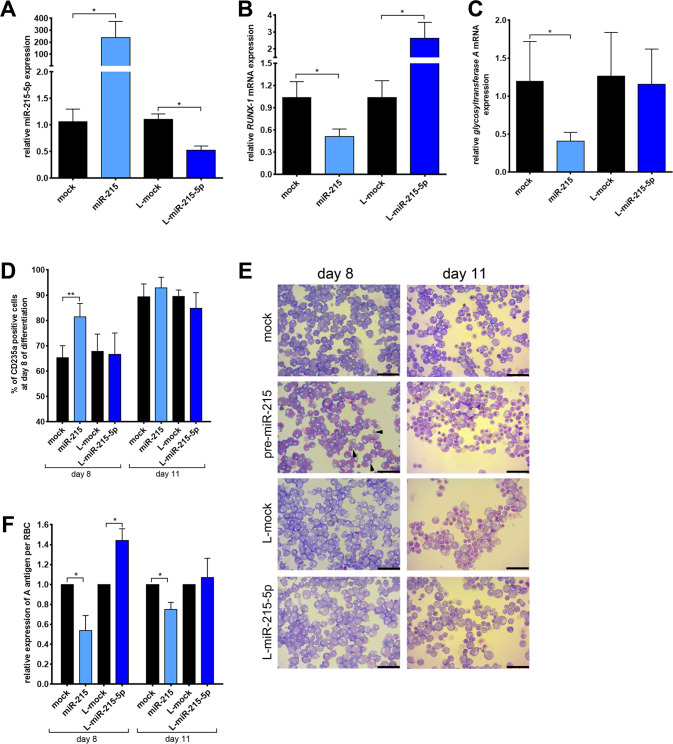
Inhibition of RUNX1 by overexpression of miR-215 leads to an accelerated erythropoiesis of CD34+ HSCs carrying BG ABO*A1.01 and downregulation of A-antigen expression. **A** Real-time PCR expression analysis of miR-215-5p in erythroid cells derived from CD34+ hematopoietic stem cells (day 8 of differentiation) transduced with pre-miR-215, or locker-miR-215-5p compared to mock controls (*n* = 3 independent experiments, genotype for all donors = ABO*A1.01/O.01.01). Relative fold changes in expression (normalized to U6) were calculated by the ΔΔCT method and values are expressed as 2-ΔΔCT to the mean values of mock controls. Real-time PCR expression analysis of *RUNX1* (**B**) and *glycosyltransferase* (**C**) mRNA in erythroid cells derived from CD34+ hematopoietic stem cells (day 8 of differentiation) transduced with pre-miR-215, or locker-miR-215-5p compared to mock controls (*n* = 3 independent experiments, genotype for all donors = ABO*A1.01/O.01.01). Relative fold changes in expression (normalized to *GAPDH*) were calculated by the ΔΔCT method and values are expressed as 2-ΔΔCT. Data are presented as mean ± standard error mean (SEM). Tests were performed two-sided. *P < 0.05 (student *t* test). **D** Amount of erythroid cells (day 8 of differentiation) after lentiviral transduction of miR-215, Locker-miR-215-5p or control (mock, L-mock) within the mCherry or GFP positive cell population measured by flow cytometry using anti-glycophorin A antibody (*n* = 4 independent experiments). **E** Giemsa staining of erythroid cells derived from CD34+ hematopoietic stem cells transduced with pre-miR-215, and locker-miR-215-5p or control (mock, L-mock). One of three representative experiments is shown (bar = 50 μm). **F** Relative amount of blood group A antigen per RBC after lentiviral transduction of miR-215 or locker-miR-215-5p within the mCherry or GFP positive and glycophorin A positive cell population compared to mock control (*n* = 4 independent experiments). **D**, **F** Data are presented as mean ± standard error mean (SEM). Tests were performed two-sided. *P < 0.05, **P < 0.01 (Mann–Whitney *U* test).

To confirm the mechanistic roles of RUNX1 and miR-215-5p with erythropoiesis and BG expression regulation we next stably overexpressed pre-miR-215 in CD34+ HSPCs using lentiviral vector gene transfer. The transduced cells were subsequently sorted for presence of the selection marker (mCherry) and overexpression was confirmed by qPCR (Fig. 7A). In a converse approach we downregulated endogenous miR-215-5p with the respective lentiviral locker plasmids, which was also confirmed by qPCR (Fig. 7A). Overexpression of pre-miR-215 leads to downregulation of both *RUNX1* and *glycosyltransferase A* mRNA. In contrast, downregulation of the endogenous miR-215-5p resulted in an upregulation of *RUNX1*, but not *glycosyltransferase A* mRNA levels (Fig. 7B, C).

Cell cultures of HSPCs transduced with pre-miR-215 showed around 20% more CD235a positive cells at day 8 of differentiation compared to mock-transduced cells, which was almost equalized until day 11 whereas downregulation of endogenous miR-215-5p did not impact erythropoiesis (Fig. 7D). We observed morphology changes that confirmed the accelerated erythropoiesis of HSPCs transduced with pre-miR-215 compared to mock transduced cells with an increase of the typical “membrane blebbing” in these cell cultures (Fig. 7E, arrowheads). Of note, the amount of BG A antigen per cell was substantially reduced (by 40–50%) upon transduction with pre-miR-215 compared to mock transduced cells and strongly increased (by 40–50%) upon downregulation of endogenous miR-215-5p (Fig. 7F).

## Discussion

The mean Hb concentrations of RBCs and hematocrit values from first-time donors of BG ABO*O and ABO*B was significantly higher when compared to BG ABO*A donors as supported by other reports [36, 37]. These differences were independently of the age and sex of the donors. However, both Hb contents and hematocrit values decreased continuously with age in men in contrast to women. The difference in Hb contents between men and women is largely due to the influence of testosterone on erythrocyte formation. As testosterone decreases, seen with age, Hb values decrease accordingly [38, 39], reflecting in part the declining contents in men as they age. In fact, it was reported that the testosterone levels inversely correlates with hepcidin serum levels and suppressed hepcidin concentrations thus allowing accelerated Hb production [40]. Hepcidin suppresses the expression of ferroportin, which plays a key role in increasing the bioavailability of iron, including for hemoglobin production [40]. Nevertheless, a direct effect of gonadotropic hormones such as testosterone has also been reported for lymphocytes [41]. Since both erythrocytes and lymphocytes arise from HSCs, it might well be that the testosterone level also directly stimulates erythropoiesis as HSPCs express functional gonadotropic hormone receptors [41].

Our findings of BG dependent Hb contents and hematocrit values can now be explained by an accelerated in vitro erythropoiesis of HSPCs from donors of BG ABO*B1.01 compared to HSPCs from the other ABO BGs. Specifically, HSPCs from individuals with BG ABO*B1.01 showed an accelerated decrease of CD34 expression, an increase in CD235a expression during erythropoiesis, and a higher enucleation rate at day 15 of differentiation. Furthermore, CFU assays illustrated the increase of lineage-specific progenitors derived from HSPCs of BG ABO*B1.01, as well as the significant higher amount of BFU-Es with three or more clusters. Therefore, we hypothesized a functional connection between the regulation of ABO BG antigens expression and erythropoiesis.

Expression of the ABO BG antigens is completed on day 7–9 of the erythroid differentiation when most cells are at the proerythroblast stage [22]. At this time point the expression of GATA-1, that plays a crucial role in the regulation of erythropoiesis [8], is strongly increased in ERPs from donors of BG ABO*B1.01 compared to donors of the other BGs. Another indicator for accelerated erythropoiesis in BG ABO*B1.01 cells is that in these cells the expression of specific miRNAs targeting TF genes involved in the inhibition of erythropoiesis, such as miR-182-5p and miR-215-5p, was increased (Supplemental Fig. S4). Previous studies elucidated the key role of miR-182-5p for the restriction of the myeloid differentiation of leukemic cells [15]. It was reported that upregulation of miR-182-5p leads to an accumulation of erythroid cells by inhibition of the TF HES-1 [15]. These data are in line with our observations, where we found a strong negative correlation between the HES-1 monomer expression and the number of proerythroblasts in ERP cultures, notably only from BG ABO*B1.01. Ross et al. described a mechanism whereby GATA-1 utilizes Ikaros and Polycomb repressive complex 2 to promote HES-1 repression as an important step in erythroid cell differentiation [42]. Further, miR-215-5p targets RUNX-1 [35], RUNX-1 interacts physically with GATA-1 to repress erythroid differentiation. Inversely, up-regulation of GATA-1 expression appears to be a feature of erythroid differentiation by repression of RUNX-1 [43]. These previous findings are in line with our observations of the negative correlation of RUNX-1 to the number of proerythroblasts in ERP cultures from BG ABO*B1.01. When erythroid cells differentiate in vitro, the ABO BG antigens expression follows a kinetic: they cannot be detected in the early phase, their expression increases gradually, and then decreases again. At the same time, expression of RUNX-1 decreases during erythroid differentiation of CD34+ cells [44, 45].

Additional genetic distinctions of the different BGs could influence the course of erythropoiesis. Specifically, there is a 43-bp repeating CBF/NF-Y enhancer present in the 5′UTR of the ABO allele. This motif has a profound effect upon transcriptional activity, with the four repeats in cells from BG ABO*B1.01, *A2.01*, *O.01* and *O.02* featuring approximately 140 times more activity than the single segment in ABO*A1.01 and *O.03* [46]. Another allelic variation in the 5′ regulatory region is the 36-bp deletion, which is specific for A1 alleles [33]. Recently, Sano et al. proposed interconnected activities for the closely located genes odorant binding protein 2B (OBP2B) and ABO in the gastric cancer cell line KATOIII [47]. They suggested that genes in this neighborhood could be co-regulated by the +22.6-kb site, which functions in an epithelial cell-specific manner [47]. Enhancer-promoter interactions can facilitated by long-range chromatin loops mediated by the chromatin architectural protein CCCTC-binding factor (CTCF) [48, 49]. Further, genetic variations in regulatory elements can act over long distances of the chromosome by influencing concordant changes in chromatin state and expression at distal sites, often by disrupting TF motifs, which can facilitate gene pair co-regulation and co-expression [50]. In erythroid cells, ABO BG antigens expression depends on an erythroid cell-specific regulatory element that, in turn, is activated by binding to GATA-1 and RUNX-1 [24, 25]. The association of ABO BG antigen expression and erythropoiesis was further supported by overexpression of pre-miR-215 in HSPCs from BG ABO*A1.01 donors. Enhanced expression of miR-215-5p leads to downregulation of both *RUNX1* and *glycosyltransferase* mRNA, which in turn results in accelerated erythropoiesis and a reduced number of A antigens per RBC. In contrast, downregulation of endogenous miR-215-5p upregulated A antigen expression on RBCs without any effect on erythropoiesis.

Recent studies in mice also demonstrated the involvement of miRNAs in the regulation of BG antigens and their impact on the regulation of erythroid differentiation [51], such supporting our hypothesis. Specifically, they showed that overexpression of miR-669m, which represses *Akap7* and *Xk* genes, inhibited late erythroblast differentiation, whereas late erythroblasts in the physiological state upregulated *Akap7* and *Xk*, suggesting that these genes are involved in late erythroblast differentiation. Furthermore, *Xk* deficiency results in McLeod syndrome and that comes with morphological and functional damage of RBCs [52]. Previously, we reported an increased expression of miR-331-3p, which targets simultaneously TF *SP1* and *glycosyltransferase* mRNA, as well as miR-1908-5p, which targets only glycosyltransferase mRNA in HSPCs and RBCs from donors of ABO*O.01.01 compared to ABO*A1.01 [22]. In our current study, we confirmed the higher expression of miR-331-3p in ERPs carrying BG ABO*O.01.01 at day 5. At day 7, there is the highest cell variability ranging from proerythroblasts to polychromatic normoblasts. This could lead to the absence of difference in the expression levels of miR-331-3p and -1908-5p at this time point. Further evidence suggesting the connection between BG antigen expression and the regulation of erythroid processes [53–55] are summarized in Table 1.

**Table 1:** Connection between BG antigen expression and the regulation of erythroid processes.

Blood group system	Involved factors	Influence on erythropoiesis	Reference
Vel blood group	SMIM1 (SNP rs1175550)	SMIM1 affects the mean Hb concentration of RBCs.	Cvejic et al. [53]
MAM blood group	EMP3	Disruption of EMP3 enhances erythroid proliferation.	Thornton et al. [55]
Lutheran blood group	KLF-1, GATA-1	Mutations in KLF-1 and GATA-1 are responsible for disease phenotypes in humans (hereditary persistence of fetal Hb, borderline HbA2, and congenital dyserythropoietic anemia).	Singleton et al. [54]
KEL blood group	miRNA-669m	Inhibition of erythroblast differentiation by suppression of X-linked Kx BG (Xk) genes, McLeod syndrome which come with morphological and functional damage of RBCs	Kotaki et al. [51]

*SMIM* Small Integral Membrane Protein, *EMP* tumor-associated epithelial membrane protein, *KLF* Kruppel-like factor, *GATA-1* GATA-binding factor.

In addition to the physiological changes, the association between ABO BG antigens expression and erythropoiesis is known for carcinomas and hematological malignancies [32, 56–58]. The association of reduction or complete deletion of A/B antigen expression with acute myeloid leukemia (AML), myelodysplastic syndrome (MDS) and myeloproliferative disorders including chronic myeloid leukemia is well documented [59]. MDS is characterized by ineffective hematopoiesis and a propensity to develop AML [60]. Besides altered DNA methylation of the ABO promoter, it was proposed that mutations in RUNX-1 might act as dominant negative inhibitors of wild-type RUNX-1, which were responsible for the decreased ABO transcription in bone marrow cells or A antigen loss on RBCs [59]. It is conceivable that the association of RUNX-1 and miR-215-5p expression could also be a causative explanation for the loss of A/B antigen expression in RUNX-1 mediated hematological malignancies.

Taken together, our study identifies the ABO BG dependent modulation of erythropoiesis by miR-182-5p, -215-5p, and -331-3p and corresponding TFs, leading to clinically relevant BG-specific differences in the hematocrit and, possibly, recovery from blood losses, too. The altered miRNA and TFs gene expressions we describe herein for the first time may be involved as well in carcinogenesis. Further, these changes could play not only an important role in the disappearance of differentiation-specific glycan antigens, such as A/B antigens, but also for the appearance of cancer-specific glycan antigens. Our data strongly suggest to further embark on the potential interference of genes involved in expression of ABO gene and its pleiotropic effects on erythropoiesis. A deeper understanding of the gene regulatory networks of erythropoiesis and their interference with BG-specific gene regulation could help to shed new light on the many clinical manifestations, where ABO BG-dependent differences for the development and progression of disease, such as myocardial infarction, thrombosis and infection, are observed.

## Supplementary information

supplemental material

## Data Availability

All data are available in the main text or Supplementary materials.
