# Supplementary material for: Novel evidence that the ABO blood group shapes erythropoiesis and results in higher hematocrit for blood group B carriers
Source: Leukemia. 2023 Feb 28;37(5):1126–37. doi: 10.1038/s41375-023-01858-4 (PMC10169640; doi:10.1038/s41375-023-01858-4)
Supplement: Supplementary file 1 — supplemental material [file 41375_2023_1858_MOESM1_ESM.pdf]

Supplemental Information for

Title: Novel evidence that the ABO blood group shapes erythropoiesis and results in higher hematocrit for blood group B carriers

Authors: Romy Kronstein-Wiedemann[1,2*†], Sarah Blecher[1*], Madeleine Teichert[2], Laura Schmidt[1], Jessica Thiel[1,2], Markus M. Müller[3], Jörn Lausen[4], Richard Schäfer[5,6], and Torsten Tonn[1,2]

Affiliations:

[1] Laboratory for Experimental Transfusion Medcine, Transfusion Medicine, Med. Faculty Carl Gustav Carus, Technische Universität Dresden; Dresden, Germany.

[2] German Red Cross Blood Donation Service North-East, Institute for Transfusion Medicine; Dresden, Germany.

[3] German Red Cross Blood Donation Service Baden-Württemberg/Hessen, Institute for Transfusion Medicine and Immunohematology; Kassel, Germany.

[4] Department of Genetics of Eukaryotes, Institute of Biomedical Genetics, University of Stuttgart; Stuttgart, Germany.

[5] German Red Cross Blood Donation Service Baden-Württemberg/Hessen, Institute for Transfusion Medicine and Immunohematology, Goethe University Hospital Frankfurt/M; Frankfurt/M, Germany.

[6] Institute for Transfusion Medicine and Gene Therapy Medical Center - University of Freiburg; Freiburg, Germany.

***Corresponding author. Email:** r.kronstein@blutspende.de

† These authors contributed equally to this work.

METHODS

Materials

Recombinant human SCF, IL-3, TPO and EPO were from Peprotech (Hamburg, Germany). Insulin solution, holo-transferrin, heparin, and hydrocortisone were from Sigma/Aldrich (Schnelldorf, Germany).

Isolation and cultivation of CD34+ hematopoietic stem/progenitor cells

Peripheral blood nuclear cells from male donors were collected by apheresis after 5 days G-CSF stimulation. From these, HSPCs were isolated by centrifugation over Biocoll (GE-Healthcare Bio-Science AB, Uppsala, Sweden) and subsequently magnetic microbead selection of CD34+ mononuclear cells (MNC's) using LS-MACS columns (Miltenyi Biotec, Bergisch Gladbach, Gemany; 96% ± 3%, purity). The cells were then cultured in IMDM (Life technologies, Darmstadt, Germany) supplemented with 1% L-glutamin (Biozym, Hessisch Oldendorf, Germany), 100 units/mL penicillin and 100 µg/mL streptomycin (Biozym), 330 µg/mL human holo-transferrin, 10 µg/mL recombinant human insulin, 2 IU/mL heparin, and 5% pooled AB serum (German Red Cross Blood Donation Service North-East, Dresden, Germany).

The expansion and differentiation procedure of the isolated CD34+ cells was previously described.[1] In the first step (day 0 to day 7 of differentiation), 1 x 10^4/mL CD34+ cells were cultured in the presence of 10^{-6} M hydrocortisone, 100 ng/mL SCF, 5 ng/mL IL-3 and 3 IU/mL EPO. In the second step (day 7 to day 11), the cells were resuspended at 10^5/mL in the presence of SCF and EPO. In the third step (day 11 to day 15), the cells were cultured with EPO alone. Cell counts were adjusted to 1 × 10^6/mL. Enucleated reticulocytes were isolated by filtration using a PALL WBF leukocyte filter (PALL Cooperation, Hampshire, United Kingdom).

Cultivation of cell lines, production of viral supernatants and lentiviral transduction of CD34+ stem cells

Cultivation of cell lines (K562, HEK293T) and production of viral supernatants were described previously.[2] Briefly, viral supernatants were produced by transient transfection of 293T cells using pLV-[hsa-miRNA] or pLV-[miR-locker] (Biosettia, San Diego, USA), containing fluorescent selection markers (mCherry and GFP, respectively), vesicular stomatitis virus envelope glycoprotein envelope plasmid (pMD2.G), and packaging plasmid pR8.94. 24 hours before lentiviral transduction CD34+ stem cells (genotype ABO*O.01/A1.01) were isolated and plated at a density of 1.5 x 10^5/well in a 12 well plate in STEMSpan SFEM (STEMCELL technologies, Grenoble, France) supplemented with 50 ng/mL SCF, 5 ng/mL IL-3 and 10 ng/mL TPO. For transduction, lentiviral supernatants at an MOI of 10-20 were added and spinoculated at 800 x *g* for 30 minutes at 32°C. Cells were cultured for 48h. Fluorescent cells were sorted in a FACSAria II (BD Bioscience, Heidelberg, Germany) three days after transduction and plated at a density of 2 x 10^4/mL in IMDM described above and cultured for analysis. Analyses were performed on day 8 and 11 of differentiation.

Flow cytometry

Cells were labeled with PE-Cy7-conjugated CD34 (clone 8G12), V450-conjucated anti-CD45 (clone HI30), PE-conjugated CD71 (clone M-A712), FITC-conjugated CD36 (clone CB38/NL07) and APC-conjugated anti-CD235a (clone GA-R2 (HIR2), BD Bioscience, Heidelberg, Germany). FMO samples (fluorescence minus one) were used as control. Analyses were performed on a FACSCanto II flow cytometer (BD Bioscience) with FlowJo Software.

DNA Isolation and ABO Genotyping

DNA was isolated from EDTA-anticoagulated blood using a commercial system (QIAamp Blood DNA Midi Kit; Qiagen, Hilden, Germany). ABO blood group genotyping was performed

using PCR with sequence specific primers (PCR-SSP) by a commercial kit (ABO-SSP; Inno-Train Diagnostik GmbH, Kronberg, Germany).

Analysis of the 5'-flanking sequence of the human ABO BG gene

Primer sequences for the mini satellite region (A, B) and the 36-bp insertion/deletion region (C, D) and the PCR amplification method are previously described[2]: sense primer (A): 5'-TATTTGGGAAGGTTTAAGCATTAG-3', antisense primer (B): 5'-TCTTGCAAG-CTTGTGGCCCAAA-3', sense primer (C): 5'-CTCCACCTCCCAAGTTCAAG- 3', and antisense primer (D): 5'-TCTCTCAGAGGAGTTTACACTG-3'. PCR amplifications were carried out in a final volume of 100 µL, containing 500 ng of genomic DNA, 1.5 mM of MgCI2, 200µM of each dNTP, 1.0 µM of each primer and 2.5 units of AmpliTaq Gold™ DNA polymerase (Applied Biosystem). Amplification was performed by one round of predenaturation at 95°C for 9 min, step-cycle mode of 30 rounds of denaturation at 94°C for 2 min, annealing at 61°C for 1.5 min, and extension at 72°C for 1.5 min, and one round of denaturation at 94°C for 2 min, annealing at 61°C for 1.5 min, and extension at 72°C for 10 min. Samples applied on 2% agarose gel were run with 0.09 M Tris boric acid buffer (pH 8.3) for 1 h at 100 V. The gels were stained with RedSafe Nucleic acid staining solution (Hiss Diagnostic, Freiburg, Germany) for the visualization of bands. Results are listed in **Supplemental Table S5**.

RNA isolation and quantitative reverse transcription polymerase chain reaction (qRT-PCR)

Total RNA was extracted from 3 x 10^6 erythroid progenitors at day 5 and 5 x 10^6 erythroid progenitors at day 7 using Pax Gene Blood miRNA kit (PreAnalytiX, QIAGEN, Hilden). Reverse transcription of mRNA was performed using Revert Aid H Minus First Strand cDNA Synthesis Kit according to the manufacturer´s directions (Fisher Scientific, Schwerte,

Germany). For qPCR the primers and probe were listed in **Supplemental Table S8**. The expression of *GATA-1* was analyzed using TaqMan assay Hs01085821_g1. (Applied Biosystems, Darmstadt, Germany). For measurement of miRNA expression, reverse transcription was performed with TaqMan MicroRNA Reverse Transcription Kit using Megaplex primer pool v3.0 and, subsequently, specific TaqMan MicroRNA Assays for miRNA-215-5p, -192-5p, -182-5p, -331-3p and -1908-5p (Applied Biosystems). Realtime PCR was performed with TaqMan Universal PCR Master Mix according to manufacturer's instruction. For miRNAs, U6 snRNA was used as the endogenous control. Gene expression was analyzed using ΔΔCT method.

Colony forming unit (CFU) assay

CD34+ stem cells were seeded (300 cells per dish) in methylcellulose plates (StemMACS CFU complete with EPO; Miltenyi). Colony-forming units were counted after 7 and 14 days of incubation at 37°C using STEMvision equipment (Stem Cell Technologies, Vancouver, BC, Canada). CFUs were examined in triplicates.

SDS gelelectrophoresis and Western blotting

5 x 10^6 cells were dissolved in Laemmli sample buffer (BioRad, Feldkirchen) on day 7 of differentiation, sonicated, boiled for 5 min and applied to SDS-PAGE. Protein was determined by Pierce 660 nm protein assay Kit according to the manufacturer´s directions (Fisher Scientific). Western Blot analysis was performed using monoclonal anti-SP1 (clone D4C3; New England Biolabs, Frankfurt/Main, Germany), monoclonal anti-RUNX1 (clone A-2; Santa Cruz Biotechnology Inc., Dallas, USA), polyclonal anti-TAL-1 (ab155195 abcam, Berlin, Germany), polyclonal anti-GATA-1 (antibodies-online, Achen, Germany), monoclonal anti-HES-1 (clone 7H11, ThermoScienific, Dreieich), monoclonal anti-p300 (clone 10D2,

ThermoScienific), monoclonal anti-EpoR (clone 3D10, antibodies-online) and monoclonal anti-GAPDH (clone FF26A; eBioscience, as loading control; 25 µg total protein per lane).

Morphological analysis

3 x 10^5 (day 0 to 11) or 5 x 10^5 cells (day 15) were suspended in PBS with Ca^{2+} and Mg^{2+} and spun down at 500 rpm without break on polysine coated glass slides. Slides were fixed with methanol and stained with 20 % Giemsa staining solution for 5 minutes. Morphology was analyzed with Zeiss Primo Vert Inverted Microscope.

Limitation of the study

We used bulk cultures of enriched CD34+ HSCPs from mobilized healthy stem cell donors in our experimental setting. It would have been also interesting to look into defined HSPC subpopulations. However, it is very difficult to obtain sufficient primary samples of CD34+ cells for all ABO blood groups to allow single cell analysis on differentiated HSPCs. Moreover, stem cells donors with BG *ABO*A/B* genotype are very rare and we failed to secure a representative source for this blood group, which is why we did not include this phenotype in our analysis.

REFERENCES

1. Giarratana MC, Rouard H, Dumont A, Kiger L, Safeukui I, Le Pennec PY*, et al.* Proof of principle for transfusion of in vitro-generated red blood cells. *Blood* 2011 Nov 10; **118**(19)**:** 5071-5079.

2. Kronstein-Wiedemann R, Nowakowska P, Milanov P, Gubbe K, Seifried E, Bugert P*, et al.* Regulation of ABO blood group antigen expression by miR-331-3p and miR-1908-5p during hematopoietic stem cell differentiation. *Stem cells (Dayton, Ohio)* 2020 Oct 1; **38**(10)**:** 1348-1362.

	No. of donors	mean + s.e.m	95% CI	p value A vs. O	p value A vs. B	p value A vs. AB	p value O vs. B	p value O vs. AB	p value B vs. AB
**age 18-27 years**				0.68	< 0.0001****	0.0001***	< 0.0001****	0.0003***	0.22
*ABO*A*	19,709	15.66 ± 0.008	15.65-15.68						
*ABO*O*	18,322	15.67 ± 0.008	15.65-15.68						
*ABO*B*	6,842	15.78 ± 0.013	15.76-15.81						
*ABO*AB*	2,882	15.75 ± 0.023	15.71-15.79						
**age 28-37 years**				0.01*	< 0.0001****	0.01*	0.0005***	0.36	0.18
*ABO*A*	11,515	15.49 ± 0.01	15.47-15.51						
*ABO*O*	10,878	15.52 ± 0.01	15.50-15.54						
*ABO*B*	4,051	15.59 ± 0.02	15.56-15.62						
*ABO*AB*	1,695	15.54 ± 0.03	15.49-15.50						
**age 38-47 years**				0.003**	< 0.0001****	0.90	0.0003***	0.19	0.0009***
*ABO*A*	7,279	15.36 ± 0.01	15.33-15.38						
*ABO*O*	7,048	15.41 ± 0.01	15.38-15.43						
*ABO*B*	2,606	15.49 ± 0.02	15.45-15.53						
*ABO*AB*	1,065	15.38 ± 0.04	15.32-15.45						
**age 48-57 years**				0.0001***	0.0001***	0.27	0.28	0.37	0.14
*ABO*A*	6,584	15.21 ± 0.01	15.18-15.23						
*ABO*O*	6,399	15.28 ± 0.01	15.25-15.30						
*ABO*B*	2,373	15.31 ± 0.02	15.27-15.35						
*ABO*AB*	977	15.25 ± 0.03	15.18-15.31						
**age ≥ 58 years**				0.49	0.0765	0.26	0.21	0.13	0.028*
*ABO*A*	2,495	15.13 ± 0.02	15.09-15.17						
*ABO*O*	2,538	15.14 ± 0.02	15.10-15.30						
*ABO*B*	900	15.19 ± 0.04	15.13-15.26						
*ABO*AB*	387	15.05 ± 0.05	14.95-15.19						

Supplemental table S1: Hemoglobin content from first time male blood donors carrying different ABO blood group antigens.

	No. of donors	mean + s.e.m	95% CI	p value A vs. O	p value A vs. B	p value A vs. AB	p value O vs. B	p value O vs. AB	p value B vs. AB
**age 18-27 years**				< 0.0001****	< 0.0001****	0.40	< 0.0001****	0.17	0.0002***
*ABO*A*	21,462	13.80 ± 0.006	13.79-13.82						
*ABO*O*	20,562	13.84 ± 0.006	13.83-13.85						
*ABO*B*	7,341	13.90 ± 0.01	13.87-13.92						
*ABO*AB*	3,189	13.82 ± 0.02	13.79-13.85						
**age 38-47 years**				< 0.0001****	< 0.0001****	0.0489*	0.001**	0.57	0.009**
*ABO*A*	9,033	13.80 ± 0.01	13.78-13.82						
*ABO*O*	8,491	13.86 ± 0.01	13.84-13.88						
*ABO*B*	3,126	13.93 ± 0.02	13.89-13.96						
*ABO*AB*	1,420	13.85 ± 0.03	13.80-13.90						
**age 28-37 years**				0.009**	< 0.0001****	0.27	0.004**	0.78	0.03*
*ABO*A*	10,740	13.81 ± 0.009	13.79-13.83						
*ABO*O*	10,792	13.85 ± 0.009	13.83-13.86						
*ABO*B*	3,806	13.90 ± 0.02	13.87-13.93						
*ABO*AB*	1,700	13.84 ± 0.02	13.79-13.88						
**age 48-57 years**				< 0.0001****	< 0.0001****	< 0.0001****	0.35	0.92	0.49
*ABO*A*	8,245	13.86 ± 0.01	13.84-13.88						
*ABO*O*	7,876	13.98 ± 0.01	13.96-14.00						
*ABO*B*	2,870	13.96 ± 0.02	13.92-13.99						
*ABO*AB*	1,238	13.98 ± 0.03	13.93-14.03						
**age ≥ 58 years**				0.0006***	0.03*	0.077	0.76	0.998	0.84
*ABO*A*	2,513	13.95 ± 0.02	13.91-13.99						
*ABO*O*	2,730	14.04 ± 0.02	14.00-14.07						
*ABO*B*	924	14.02 ± 0.03	13.96-14.08						
*ABO*AB*	399	14.05 ± 0.05	13.95-14.14						

Supplemental table S2: Hemoglobin content from first time female blood donors carrying different ABO blood group antigens.

	No. of donors	mean + s.e.m	95% CI	p value A vs. O	p value A vs. B	p value A vs. AB	p value O vs. B	p value O vs. AB	p value B vs. AB
**age 18-27 years**				0.087	0.0001***	0.55	< 0.0001****	0.16	0.069
*ABO*A*	12,064	0.4584 ± 0.0002	0.4580-0.4589						
*ABO*O*	11,092	0.4581 ± 0.0002	0.4576-0.4585						
*ABO*B*	3,689	0.4605 ± 0.0004	0.4597-0.4613						
*ABO*AB*	1,534	0.4593 ± 0.0007	0.4580-0.4607						
**age 28-37 years**				0.67	0.0037**	0.061	0.01*	0.099	0.93
*ABO*A*	4,683	0.4560 ± 0.0004	0.4552-0.4567						
*ABO*O*	4,547	0.4562 ± 0.0004	0.4554-0.4570						
*ABO*B*	1,437	0.4583 ± 0.0007	0.4570-0.4597						
*ABO*AB*	596	0.4579 ± 0.001	0.4557-0.4600						
**age 38-47 years**				0.85	0.03*	0.26	0.04*	0.21	0.019*
*ABO*A*	2,984	0.4535 ± 0.0005	0.4526-0.4545						
*ABO*O*	2,878	0.4539 ± 0.0005	0.4529-0.4548						
*ABO*B*	942	0.4556 ± 0.0009	0.4540-0.4573						
*ABO*AB*	418	0.4521 ± 0.001	0.4495-0.4548						
**age 48-57 years**				0.18	0.18	0.053	0.68	0.01*	0.01*
*ABO*A*	2,348	0.4520 ± 0.0006	0.4509-0.4531						
*ABO*O*	2,371	0.4530 ± 0.0006	0.4519-0.4541						
*ABO*B*	754	0.4538 ± 0.001	0.4517-0.4558						
*ABO*AB*	307	0.4486 ± 0.002	0.4454-0.4519						
**age ≥ 58 years**				0.38	0.017*	0.34	0.068	0.64	0.48
*ABO*A*	864	0.4492 ± 0.001	0.4472-0.4511						
*ABO*O*	855	0.4505 ± 0.001	0.4484-0.4525						
*ABO*B*	257	0.4535 ± 0.002	0.4499-0.4571						
*ABO*AB*	133	0.4515 ± 0.002	0.4468-0.4562						

Supplemental table S3: hematokrit values from first time male blood donors carrying different ABO blood group antigens.

	No. of donors	mean + s.e.m	95% CI	p value A vs. O	p value A vs. B	p value A vs. AB	p value O vs. B	p value O vs. AB	p value B vs. AB
**age 18-27 years**				0.0002***	<0.0001****	0.40	0.005**	0.35	0.0098**
*ABO*A*	12,751	0.4109 ± 0.0002	0.4105-0.4113						
*ABO*O*	12,139	0.4121 ± 0.0002	0.4116-0.4125						
*ABO*B*	3,903	0.4133 ± 0.0004	0.4126-0.4141						
*ABO*AB*	1,622	0.4117 ± 0.0006	0.4105-0.4130						
**age 28-37 years**				<0.0001****	0.0003***	0.19	0.53	0.39	0.25
*ABO*A*	4,143	0.4123 ± 0.0004	0.4115-0.4130						
*ABO*O*	4,062	0.4146 ± 0.0004	0.4138-0.4154						
*ABO*B*	1,283	0.4152 ± 0.0007	0.4138-0.4165						
*ABO*AB*	580	0.4133 ± 0.001	0.4113-0.4153						
**age 38-47 years**				0.0007***	0.03*	0.12	0.90	0.97	0.96
*ABO*A*	3,522	0.4126 ± 0.0004	0.4117-0.4134						
*ABO*O*	3,484	0.4148 ± 0.0004	0.4139-0.4156						
*ABO*B*	1,023	0.4148 ± 0.0008	0.4132-0.4164						
*ABO*AB*	481	0.4151 ± 0.001	0.4126-0.4175						
**age 48-57 years**				0.016*	0.004**	0.23	0.92	0.01*	0.47
*ABO*A*	2,956	0.4169 ± 0.0005	0.4159-0.4179						
*ABO*O*	2,911	0.4182 ± 0.0005	0.4173-0.4192						
*ABO*B*	924	0.4196 ± 0.0009	0.4179-0.4214						
*ABO*AB*	347	0.4180 ± 0.001	0.4152-0.4207						
**age ≥ 58 years**				0.51	0.16	0.01*	0.33	0.025*	0.15
*ABO*A*	792	0.4190 ± 0.001	0.4171-0.4209						
*ABO*O*	873	0.4201 ± 0.0009	0.4183-0.4219						
*ABO*B*	261	0.4227 ± 0.0019	0.4189-0.4265						
*ABO*AB*	90	0.4265 ± 0.0029	0.4207-0.4323						

Supplemental table S4: hematokrit values from first time female blood donors carrying different ABO blood group antigens.

Genotype	Total sample number	Numer of 43 bp repeats		36 bp deletion/insertion	
		**1**	**4**	**deletion**	**insertion**
ABO*A1.01/A1.01	2	2	0	2	0
ABO*A1.01/O.01.01	15	15	15	15	15
ABO*A2.01/O.01.01	1	0	1	0	1
ABO*O.01.01/O.01.01	13	0	13	0	13
ABO*B1.01/B1.01	3	0	3	0	3
ABO*B1.01/O.01.01	11	0	11	0	11
ABO*A1.01/B.1.01	4	4	4	4	4
ABO*A2.01/B.1.01	1	0	1	0	1

Supplemental Table S5. Analysis of the 5'-flanking sequence of the human ABO blood group gene.

BG	Number of total colonies								Percentage of the type of colonies within the total number of colonies (%)					
**d 7**	**total**	**p-value**	**erythroid**	**p-value**	**myeloid**	**p-value**	**mixed**	**p-value**	**erythroid**	**p-value**	**myeloid**	**p-value**	**mixed**	**p-value**
ABO*A (n=17)	64.1±6.7		17.2±1.64		45.7±5.34		3.1±0.62		28.4±2.2		65.4±2.7		5.6±1.0	
ABO*O (n=13)	86.0±9.1	0.10	**25.3±2.83**	**0.042***	57.1±7.08	0.211	3.6±0.53	0.28	31.1±3.5	0.63	64.7±3.9	0.83	4.5±0.6	0.40
ABO*B (n=14)	**93.8±9.1**	**0.01***	24.8±3.51	0.061	**66.2±7.37**	**0.049***	2.9±0.46	0.90	27.2±2.8	0.48	68.8±3.0	0.37	**3.1±0.4**	**0.049***
ABO*AB (n=4)	**111.3±10.5**	**0.003****	**27.3±3.33**	**0.015***	**79.3±6.66**	**0.01***	4.8±0.76	0.15	24.3±1.1	0.55	71.4±1.4	0.39	4.2±0.4	0.48
**d 14**	**total**	**p-value**	**erythroid**	**p-value**	**myeloid**	**p-value**	**mixed**	**p-value**	**erythroid**	**p-value**	**myeloid**	**p-value**	**mixed**	**p-value**
ABO*A (n=17)	72.8±6.1		21.7±1.64		45.3±4.27		5.8±1.1		30.9±2.1		59.8±2.5		7.9±1.1	
ABO*O (n=13)	**96.8±5.6**	**0.01***	**29.3±2.56**	**0.02***	**59.5±3.99**	**0.044***	8.0±0.82	0.052	30.2±2.1	0.91	61.7±2.3	0.66	8.2±0.7	0.54
ABO*B (n=14)	**99.5±10.7**	**0.03***	26.8±4.01	0.25	**66.6±7.36**	**0.017***	6.1±0.92	0.46	26.5±2.6	0.105	67.1±3.1	0.067	5.9±0.7	0.22
ABO*AB (n=4)	**110.8±9.2**	**0.01***	28.7±4.01	0.195	**74.8±5.44**	**0.005****	7.3±1.16	0.19	25.7±2.4	0.399	67.8±2.4	0.14	6.5±0.6	0.54

Supplemental Table S6. Impact of the ABO blood group on the in vitro differentiation and proliferation potential of HSPCs at day 7 and day 14. Data are presented as mean ± standard error mean (SEM). Tests were performed two-sided (Mann-Whitney-U test). *P*-values indicate significant differences of the different blood group types compared with blood group type A (consensus). The bold entries represent significant differences.

	Blood group phenotype								
	***ABO*A1.01* (n = 18)**			***ABO*O.01.01* (n = 13)**			***ABO*B1.01* (n = 14)**		
**correlation**	**r**	**R^2**	***p value***	**r**	**R^2**	***p value***	**r**	**R^2**	***p value***
CD34+ cell number d5/ proerythroblast d5	**-0.93**	**0.87**	**<0.0001**	**-0.85**	**0.72**	**0.0002**	**-0.78**	**0.61**	**0.0011**
CD34+ cell number d5/ GATA-1 d7	-0.47	0.22	0.05	-0.17	0.03	0.581	**0.67**	**0.45**	**0.008**
CD34+ cell number d5/ RUNX1 d7	0.12	0.02	0.626	-0.03	0.001	0.913	**0.86**	**0.74**	**<0.0001**
CD34+ cell number d5/ TAL1 d7	-0.04	0.001	0.885	-0.29	0.08	0.340	**0.80**	**0.63**	**0.0007**
CD34+ cell number d5/ HES1 d7	0.005	0.00	0.985	-0.02	0.0006	0.938	**0.82**	**0.68**	**0.0003**
CD34+ cell number d5/ SP1 d7	-0.02	0.0003	0.943	0.07	0.004	0.830	**0.73**	**0.54**	**0.0029**
CD34+ cell number d5/ EpoR d7	0.14	0.02	0.588	-0.26	0.07	0.398	**0.53**	**0.29**	**0.0493**
CD34+ cell number d5/ p300 d7	0.25	0.06	0.310	-0.17	0.03	0.591	0.37	0.14	0.187
CD34+ cell number d5/ GR d7	**-0.59**	**0.34**	**0.011**	**-0.64**	**0.41**	**0.018**	-0.30	0.09	0.306
proerythroblast number d5/ GATA-1 d7	0.40	0.16	0.098	0.07	0.005	0.824	**-0.69**	**0.47**	**0.0069**
proerythroblast number d5/ RUNX1 d7	-0.18	0.031	0.484	-0.19	0.04	0.532	**-0.59**	**0.35**	**0.025**
proerythroblast number d5/ TAL1 d7	0.03	0.0008	0.911	0.28	0.08	0.348	**-0.61**	**0.37**	**0.02**
proerythroblast number d5/ HES1 d7	0.07	0.004	0.795	-0.11	0.013	0.713	**-0.77**	**0.59**	**0.0013**
proerythroblast number d5/ SP1 d7	0.09	0.009	0.713	-0.07	0.006	0.808	**-0.67**	**0.44**	**0.0095**
proerythroblast number d5/ EpoR d7	-0.14	0.019	0.589	-0.005	0.00	0.988	**-0.57**	**0.32**	**0.035**
proerythroblast number d5/ p300 d7	-0.20	0.039	0.431	-0.07	0.005	0.815	-0.27	0.07	0.353
proerythroblast number d5/ GR d7	**0.65**	**0.42**	**0.0037**	**0.64**	**0.41**	**0.018**	-0.13	0.02	0.368
GATA-1 d7/ GR d7	**0.75**	**0.56**	**0.0003**	0.05	0.002	0.881	-0.20	0.04	0.505

Supplemental Table S7. Correlation between the percentage of CD34+ HSCs or proerythroblasts at day 5 with the protein expression levels of certain transcription factors at day 7. r = Pearson correlation coefficient, R^2 = coefficient of determination. Significant values are marked in bold.

Gene	Primer direction	Primer sequence 5‘ → 3‘
HES-1	forward	TTG GAG GCT TCC AGG TGG TA
	reverse	GCC CCG TTG GGA ATG AG
	probe	FAM-CTC CCG ATG GCC AGT T-TAMRA
RUNX1	forward	TGC CAG CGG CAT GAC A
	reverse	TCG GGT GCC GTT GAG AGT
	probe	FAM-CCC TCT CTG CAG AAC T-TAMRA
TAL1	forward	ACC GAT CCC AGT TGG AGG AT
	reverse	TCC CCC TTT TTC GCT GAG A
	probe	FAM-CAC CGC AGC GTA AC-TAMRA
EpoR	forward	CTG GTG GCA GTG TGG ACA TAG T
	reverse	CTT CGA GGC CAA AGC AGA TG
	probe	FAM-CTC AGA AGC ATC CTC CT-TAMRA
p300	forward	CAA CAG AGC AGT CCT GGA TTA GG
	reverse	TTG ACT GCG TAG GAC CCT GAT
	probe	FAM-CAC AGG CAG GCT TGA-TAMRA
SP1	forward	GTG CCG CTC CCA ACT T
	reverse	TGT GGG ATT ACT TGA TAC TGA ATA TTA GG
	probe	FAM-ACC AGC AAG TTC TGA CAG G-TAMRA
glycosyltransferase A	forward	GGT CTA CCC CCA GCC AAA G
	reverse	GGT CAC CAC GAG GAC ATC CT
	probe	FAM-TGC TGA CAC CGT GTA GG-TAMRA
GAPDH	forward	AGG GCT GCT TTT AAC TCT GGT AA
	Reverse	CAT GGG TGG AAT CAT ATT GGA AC
	probe	FAM-TGT TGC CAT CAA TGA CCC CTT CAT TG-TAMRA

Supplemental Table S8. Primers and probes for amplification by qPCR.

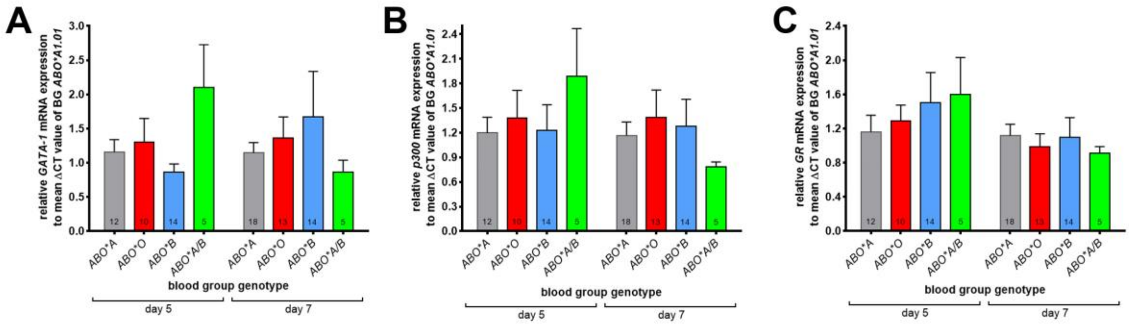

Supplemental Figure S1: Quantitative PCR of different transcription factors at day 5 and day 7 of erythroid differentiation. Relative expression of *GATA-1* **(A)**, *p300* **(B)**, and *glucocorticoid receptor* **(C)** to the mean value of ΔCT from cells carrying BG *ABO*A1.01*. Relative fold changes in expression (normalized to *GAPDH*) were calculated by the ΔΔCT method and values are expressed as $2^{-\Delta\Delta CT}$. The number of analyzed donors was indicated in the corresponding bars. Data are presented as mean ± SEM. Tests were performed two-sided.

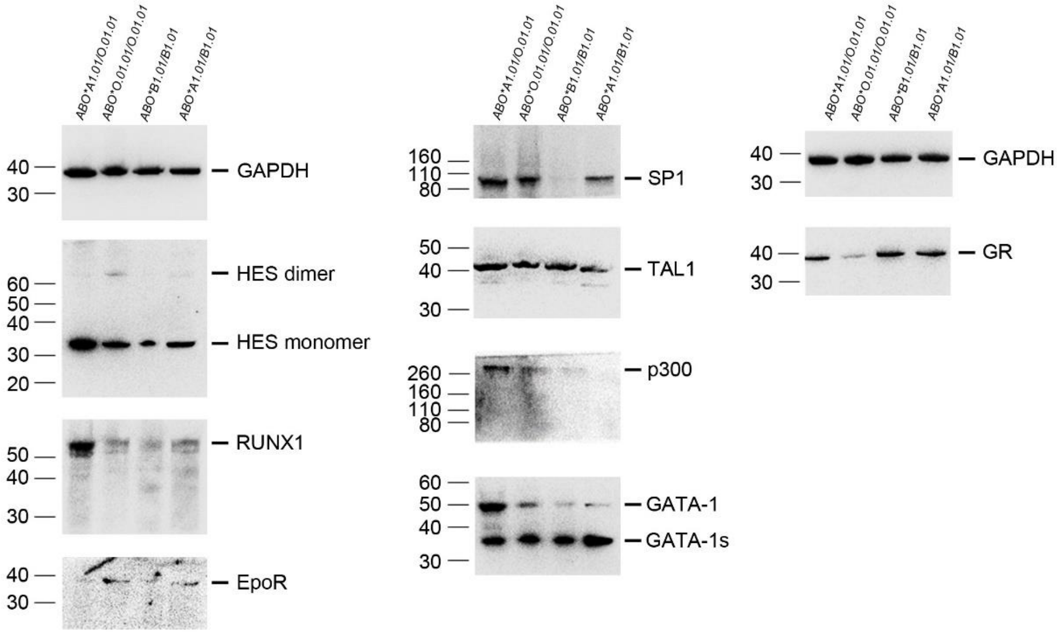

Supplemental Figure S2: Western blot analysis of several transcription factors from erythroid cells derived from CD34+ hematopoietic stem cells at day 7 of differentiation. One representative experiment of each blood group phenotype is shown. Western Blot analysis was performed using monoclonal anti-SP1, anti-RUNX1, anti-HES-1, anti-p300, anti-EpoR, anti-glucocorticoid receptor, polyclonal anti-TAL-1, anti-GATA-1, and monoclonal anti-GAPDH as loading control.

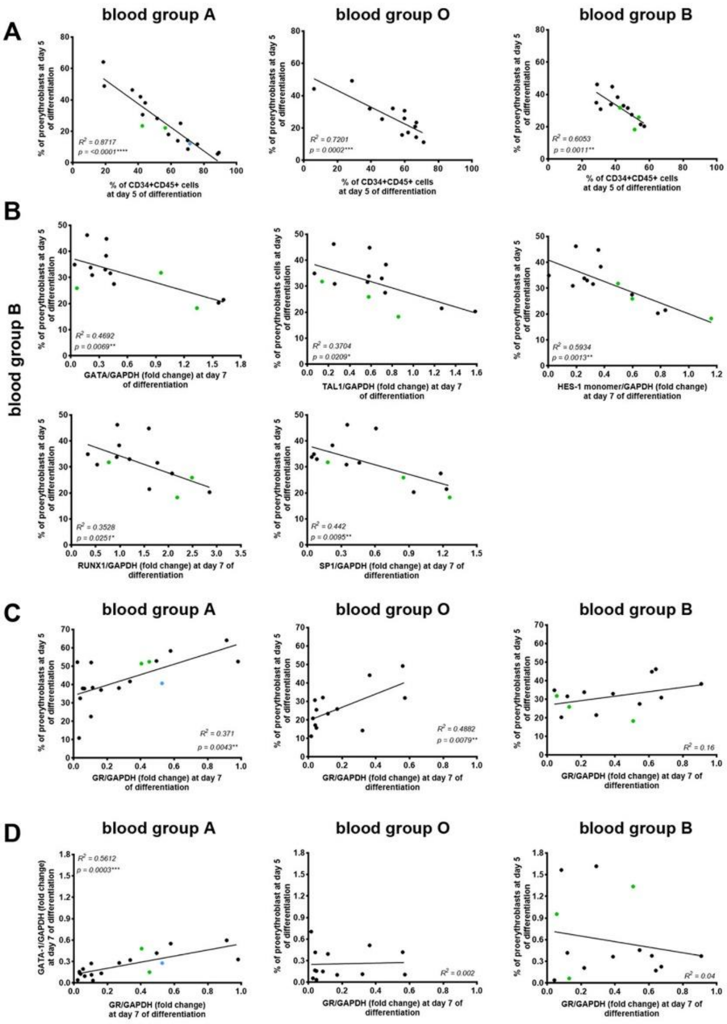

Supplemental Figure S3: The percentage of CD34+ HSCs and expression of certain transcription factors are correlating only during differentiation of cell carrying BG *ABO*B1.01*. (A) Correlation between the percentage of CD34+ HSCs and the percentage of proerythroblasts at day 5 of differentiation. **(B)** Correlation between the percentage of CD34+ HSCs carrying BG *ABO*B1.01* at day 5 of differentiation and the expression of GATA-1, TAL1, HES1, RUNX1, and SP1 at day 7 of differentiation. (C) Correlation between the percentage of CD34+ HSCs and the expression of GR at day 7 of differentiation depending on the BG phenotype. Each dots represents one donor. Green dots indicate homozygous donors and the blue dots indicate the donor with BG *ABO*A2.01*. R^2 = correlation coeffictient. Tests were performed two-sided. **P* < 0.05; ***P* < 0.01; *** P < 0.001.

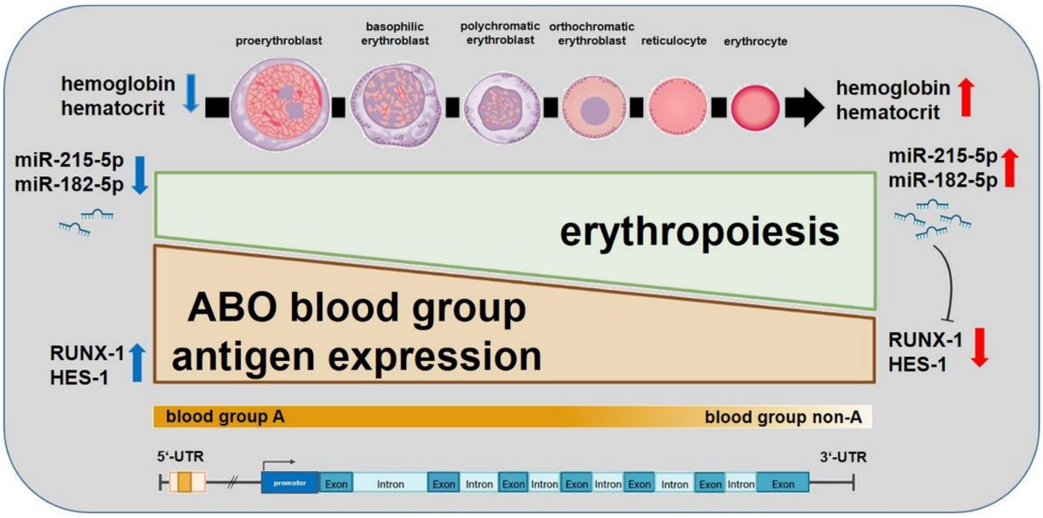

Supplemental Figure S4: The mechanism proposed in this study. Different miRNA repertoires/compositions of hematopoietic stem cells in dependence of the underlying ABO blood group interacts with erythroid lineage specific transcription factors and thus affects the velocity and yield of hematopoietic differentiation.
